# What Do Older Adults Want From Social Robots and Artificial Intelligence? A Product-Oriented Perspective

**DOI:** 10.1093/geroni/igaf122.3869

**Published:** 2025-12-31

**Authors:** Nicole Long-ki Fung, Yueyuan Zheng, Bertram Shi, Janet Hui-wen Hsiao, Jean Woo

**Affiliations:** The Chinese University of Hong Kong, Hong Kong, Hong Kong, China (People’s Republic); Hong Kong University of Science and Technology, Hong Kong, Hong Kong, China (People’s Republic); Hong Kong University of Science and Technology, Hong Kong, Hong Kong, China (People’s Republic); Hong Kong University of Science and Technology, Hong Kong, Hong Kong, China (People’s Republic); The Chinese University of Hong Kong, Hong Kong, Hong Kong, China (People’s Republic)

## Abstract

The global aging population is placing increasing pressure on healthcare and social resources. Many have turned to technology to offset the impact of manpower shortage in eldercare. Social robots and artificial intelligence hold promising potential in enhancing older adults’ well-being and quality of life. While older adults generally express positive attitudes towards these technologies, adoption rates remain relatively low, with older adults citing concerns about design, usability, and relevance. Existing technological acceptance models emphasize the effects of individual factors or subjective evaluations (e.g., perceived usefulness) on product acceptance, offering limited guidance on the product attributes that foster positive perceptions among older adults. This study adopted a product-oriented perspective to understand older adults’ preferences for social robots and artificial intelligence. We searched through five databases, including PsycINFO, Web of Science, IEEE Xplore Digital Library, Scopus, and ACM Digital Library, and identified 243 relevant articles published until January 2025. We extracted 22 product features deemed desirable or undesirable by older adults and organized them according to self-determination theory and socioemotional selectivity theory. Older adults valued features that support their autonomy (e.g., customization), relatedness (e.g., facilitating social connections), and competence (e.g., providing living assistance), as well as features serving emotional regulation (e.g., providing entertainment), pro-closeness (e.g., grandchildren-like voices), and self-relevance goals (e.g., offering culturally specific features like dialects). Our synthesis highlights the psychological needs and motivational priorities underlying older adults’ technology preferences. These findings provide actionable insights for designing human-centered technologies that are more likely to be accepted and adopted by older adults.

